# The Significance of CXCL1 and CXCR1 as Potential Biomarkers of Colorectal Cancer

**DOI:** 10.3390/biomedicines11071933

**Published:** 2023-07-07

**Authors:** Marta Łukaszewicz-Zając, Monika Zajkowska, Sara Pączek, Agnieszka Kulczyńska-Przybik, Kamil Safiejko, Marcin Juchimiuk, Leszek Kozłowski, Barbara Mroczko

**Affiliations:** 1 Department of Biochemical Diagnostics, Medical University of Bialystok, 15-269 Bialystok, Poland; marta.lukaszewicz-zajac@umb.edu.pl (M.Ł.-Z.); barbara.mroczko@umb.edu.pl (B.M.); 2 Department of Biochemical Diagnostics, University Hospital of Bialystok, 15-269 Bialystok, Poland; sara.paczek@uskwb.pl (S.P.); agnieszka.kulczynska-przybik@umb.edu.pl (A.K.-P.); 3 Department of Neurodegeneration Diagnostics, Medical University of Bialystok, 15-269 Bialystok, Poland; 4 Bialystok Oncology Centre, 15-027 Bialystok, Poland; kamil.safiejko@gmail.com (K.S.); jumedica.onkologia@gmail.com (M.J.); leszek@kozlowski.pl (L.K.)

**Keywords:** colorectal cancer, chemokines, biomarker

## Abstract

The CXCL1/CXCR2 and CXCL8-CXCR1/CXCR2 axes are under intensive investigation as they appear to regulate the progression and invasion of colorectal cancer (CRC). Growing evidence demonstrates the elevated expression of these proteins in CRC. However, a majority of relevant studies have been performed on CRC tissues using immunohistochemical techniques. Our study is the first to evaluate the diagnostic significance of serum CXCL1 and CXCR1 levels in CRC patients in comparison to well-established tumor markers, such as the carcinoembryonic antigen (CEA), and markers of inflammation, such as C-reactive protein (CRP). Thus, the aim of our study was to assess whether circulating serum levels of CXCL1 and CXCR1 might be candidates for novel biomarkers in the diagnosis and progression of CRC. The study was performed on 76 subjects, including patients with CRC and healthy volunteers as a control group. Serum concentrations of CXCL1, CXCR1, and the classical tumor marker (CEA) were measured using immunoenzyme assays, while CRP levels were assessed with the immunoturbidimetric method. Serum CXCL1 levels were statistically significantly increased in CRC patients when compared to healthy subjects, and similar results were found for CEA and CRP levels. The percentage of elevated concentrations of CXCL1 and CXCR1 was higher than that of the classical tumor biomarker and increased in the combined measurement of these proteins with CEA. In addition, among all proteins tested, serum CXCL1 seems to be the best indicator in the differentiation between CRC patients with nodal involvement and patients without the presence of lymph node metastasis. Our preliminary results indicate the role of serum CXCL1 and CXCR1 in the diagnosis of CRC, particularly in the combined measurement with CEA.

## 1. Introduction

Colorectal cancer (CRC) is the second-most frequent malignancy worldwide. The global burden of this neoplasm is expected to increase, with 2.2 million new cases and 1.1 million annual deaths by 2030 [1,2,3]. Colorectal carcinomas are classified as sporadic, familial, or inherited, based on the origin of the mutation [4,5]. The most significant prognostic factor in CRC is the tumor stage at the time of diagnosis. However, the disease is commonly detected based on the presence of symptoms that appear in advanced stages of tumor development [6]. Thus, due to the high mortality and morbidity of CRC, there is an urgent need to establish novel diagnostic tools for the detection of early-stage CRC.

CRC develops from noncancerous polyps which may transform into early adenoma, and may subsequently become malignant tumors [7,8,9,10,11]. It has been proven that prolonged chronic inflammation is a key factor in the pathogenesis of many malignancies, including CRC, due to the promotion of the synthesis of proinflammatory mediators, e.g., chemokines, within the tumor microenvironment. Some clinical investigations have indicated that leukocytes and cytokines, including selected chemokines, as well as tumor cells present within the tumor microenvironment (TME), may act as mediators of inflammatory states and stimulate prolonged inflammation [8]. The interaction between various inflammatory mediators within the TME might facilitate the migration of malignant cells through the stroma and angiogenesis, thus promoting tumor progression. Despite advances in research regarding the impact of inflammation on the development of malignant disease, the role of inflammatory mediators in tumor progression has not been fully elucidated [9]. A number of investigations, most of which used the immunohistochemistry method, explored the clinical significance of selected inflammatory mediators, including chemokines, as potential biomarkers for CRC [12,13,14]. It has been proven that several chemokines, such as CCL2, CCL3, CCL4, CXCL1, CXCL8, and CXCL10, and their specific receptors, including CXCR1, CXCR2, CXCR3, CXCR4 and CXCR7, are highly expressed in colon cancer cells [6,15].

The diagnosis of CRC is a complex process involving imaging modalities and laboratory tests. The carcinoembryonic antigen (CEA) is a well-established marker for this malignancy, although its utility in screening for the disease is not satisfactory because of its low diagnostic sensitivity, particularly in early-stage CRC [10,11]. Therefore, new noninvasive biomarkers for the early detection of CRC are crucial to improving the management of patients with this disease, especially in the early stages. Moreover, there are review publications in the literature which discuss the usefulness of many parameters in the diagnosis of CRC. The authors of these papers often emphasize the necessity of identifying easily measurable, non-invasive and cost-effective biomarkers for CRC screening and treatment [16].

C-X-C motif ligand 1 (CXCL1) belongs to a family of chemotactic cytokines that are synthesized by leukocytes and different tissue cells. Thus, they are regulators of leukocyte migration in, e.g., inflammatory processes [17,18,19,20]. CXCL1 binds to its two cognate G-protein-coupled receptors, such as CXCR2 (C-X-C motif chemokine receptor 2), to promote cell migration, adhesion and chemotaxis. A growing body of evidence has revealed that various C-X-C motif chemokines and their specific receptors may facilitate the migration, proliferation, invasion, angiogenesis and metastasis of tumor cells in numerous malignancies including CRC [17,18,19,20,21,22,23]. Interestingly, the available bioinformatics data and some of the works on CXCL1 have proven its high expression in CRC tissues and cell lines (SW837, SW480, CaCO2, and HT29). In view of this, it could be reasoned that the high expression of CXCL1 might have a positive effect on CRC development. Based on this information, we decided to check the usefulness of this parameter when assessing its concentrations in the most popular and easily available biological materials. Using cell models, it has also been proven that silencing CXCL1 inhibits cell proliferation, migration and invasion, as well as promotes cell apoptosis in CRC [24]. Thus, the CXCL1/CXCR2 axis plays a significant role in CRC development.

Some clinical investigations have indicated that the CXCL8-CXCR1/2-mediated signaling pathway is also associated with inflammation and indirectly with cancer development. CRC cells are able to regulate CXCL8 in an autocrine manner and increase the expression of CXCR1 and CXCR2, which contributes to tumor development through the invasion, dissemination and metastasis of cancer cells [6,25,26,27].

In our most recent investigations, we assessed the diagnostic and prognostic significance of the receptor of CXCL1 (CXCR2) as well as its ligand (CXCL8) in CRC patients using the enzyme-linked immunosorbent assay (ELISA) method [28,29]. Based on our latest results, we concluded that serum CXCL-8 might be a better biochemical marker candidate in the diagnosis of CRC than the classical tumor marker (CEA). Recently, we also published a review where the potential significance of CXCL1 and CXCL8 in the diagnosis and progression of CRC, as well as that of their specific receptors CXCR2 and CXCR1, was described. Therefore, as a continuation of our previous research, in the present investigation, we assess whether another ligand of CXCR2, such as CXCL1, as well as a second receptor of CXCL8, such as CXCR2, might be used as potential biomarkers for CRC. Some clinical investigations have demonstrated that CXCL1 expression is higher in CRC tissue in comparison to a healthy control and is significantly correlated with a larger tumor size, more advanced tumor stage, greater depth of invasion and presence of lymph node metastasis, larger tumor diameter, and higher CEA levels [30,31,32,33,34,35,36,37,38]. The majority of studies investigating this issue have examined tissue samples of CRC to determine the expression of the tested chemokine in CRC cells. To the best of our knowledge, the present study is the first to demonstrate the clinical significance of serum CXCL1 and CXCR1 concentrations in relation to the established first-line tumor marker for CRC—CEA. It also examines the relationship between the concentrations of CXCL1, CXCR1, and C-reactive protein (CRP), a marker of inflammation in CRC patients, which allows us to better understand the link between inflammation and tumor development.

## 2. Materials and Methods

The present, retrospective study included 76 subjects: 46 CRC patients and 30 healthy volunteers. CRC patients were diagnosed in Bialystok Oncology Centre (Poland). The clinical diagnosis of CRC was confirmed based on the microscopic examination of the material obtained during biopsy and/or surgery. All cancer cases were staged using the TNM (tumor–node–metastasis) classification. The characteristics of the CRC patients (aged 33–79 years) included in the study are presented in Table 1. The control group comprised 30 healthy volunteers, matched by age and sex, in whom inflammatory or neoplastic diseases were excluded (17 men and 13 women, aged 34–80 years) and who were recruited from hospital volunteer organizations. All samples were anonymized.

Patients with CRC included in the study were divided into groups based on the following criteria: tumor stage (I, II and III + IV), depth of tumor invasion (T1 + T2, T3 and T4), presence of lymph node metastasis (node-negative (N0) and node-positive (N1)), distant metastasis (M0 and M1), differentiation of the tumor (G1, G2 and G3) and histological subtype of the tumor (adenocarcinomas and mucinous adenocarcinomas).

The following exclusion criteria were applied: no active infections and a lack of symptoms of an infection, the absence of comorbidities that could affect protein concentrations (respiratory diseases and digestive tract diseases), and the absence of systemic diseases such as lupus and rheumatoid arthritis. All study participants had a BMI within the healthy weight range in order to exclude obesity-related inflammatory factors. 

The study was conducted in accordance with the Declaration of Helsinki. It was approved by the Local Ethics Committee (R-I-002/65/2017) of the Medical University of Bialystok (Poland). All participants were informed about the purpose of the study. All participants gave written informed consent.

Blood samples were collected from patients between August 2019 and March 2020 (Sarstedt, Nümbrecht, Germany) prior to treatment and stored at −80 °C until analysis (May 2020). Serum CXCL1 and CXCR1 levels were measured using enzyme-linked immunosorbent assay kits (ELISA) (Quantikine ELISA, Human CXCL1/GROα Immunoassay, Abingdon, R&D systems, UK; CXCR1 Immunoassay, EIAab, Wuhan, China) according to the manufacturers’ instructions. Concentrations of the established tumor marker (CEA) were examined by the chemiluminescent microparticle immunoassay (CMIA) method (Abbott, Green Oaks, IL, USA) using the ARCHITECT 8200 ci, while serum CRP levels were measured by the immunoturbidimetric method (Abbott, Green Oaks, IL, USA) using the ARCHITECT 8200 ci. Youden’s index was used to select optimal predicted probability cut-off values. The reference cut-off values were as follows: 118.91 pg/mL for CXCL1; 0.03 ng/mL for CXCR1; 2.24 ng/mL for classical tumor marker CEA and 2.15 mg/l for CRP.

### Statistical Analysis

In the preliminary statistical analysis (χ^2^ test), serum levels of CXCL-1, CXCR1, CRP and CEA did not follow a normal distribution, and therefore, nonparametric statistical analyses were used. To compare two groups, the Mann–Whitney test was employed, while the Kruskal–Wallis test was used for three or more groups. Moreover, if significant differences were found, the post hoc Dwass–Steele–Critchlow–Fligner test was employed, while the Spearman rank correlation test was used for correlation analyses. Diagnostic sensitivity and specificity, accuracy, predictive values for positive (PPV) and negative (NPV) results, and the AUC were calculated to assess the diagnostic importance of all analyzed proteins. Differences were considered statistically significant when *p* < 0.05. IBM SPSS Statistics 20.0 was used for statistical analysis, whereas Microsoft Office Excel was employed to calculate diagnostic parameters. Furthermore, logistic regression was used to assess the strength of the association between various risk factors and CRC. Firstly, univariate logistic regression models were used to assess the relationship between each variable and CRC risk. Secondly, variables where *p* < 0.05 were introduced into the multivariate model. Finally, the least significant variables were removed from the model in a stepwise manner based on the Wald statistic. None of the patients were excluded at any stage of the analysis. Reporting recommendations for tumor marker prognostic studies (REMARK) were added and can be found in Supplementary Table S1.

## 3. Results

**Figure 1:**
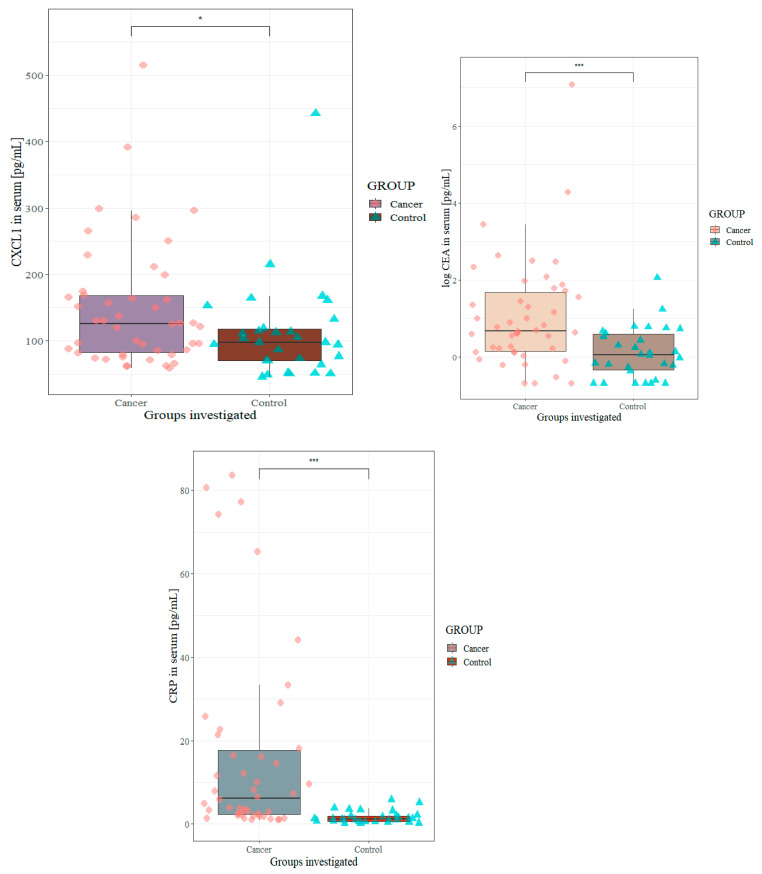
Serum concentrations of CXCL1, CEA and CRP in CRC patients in comparison to healthy controls; * statistically significant when *p* < 0.05; *** statistically significant when *p* < 0.001. CEA, carcinoembryonic antigen; CRP, C-reactive protein; CXCL1, C-X-C motif ligand 1.

Serum CXCL1 concentrations were significantly higher in CRC patients in comparison to healthy controls (*p* = 0.034), and were similar to those of the well-established biomarker CEA (*p* < 0.001) and the indicator of inflammation CRP (*p* < 0.001) (Figure 1). The concentrations of CXCR1 were lower in CRC patients than in the control group; however, this difference was not significant.

The analysis of the relationship between the CXCL1 concentration and the tumor stage based on the TNM classification revealed that serum concentrations of this protein were higher in the early stages of cancer (stage I and II) in comparison to advanced CRC (stage III + IV), which is similar for CXCR1. The highest levels of CEA were found in patients with stages III + IV of CRC. Moreover, CEA concentrations were significantly higher in patients with stage II (*p* = 0.032) and stage III + IV (*p* = 0.009) cancer in comparison to healthy subjects. In addition, CRP levels were the highest in stage II cancer, and these differences were statistically significantly higher when compared to the control group (*p* < 0.001) (Table 2).

When we analyzed the association between concentrations of the tested proteins and the clinicopathological characteristics of the tumor, such as the depth of tumor invasion (T factor) and the presence of lymph nodes (N factor) and distant metastases (M factor), CXCL1 concentrations were the lowest in the T4 subgroup than in the T1 + 2 and T3 patients (Table 3). Serum levels of the classical tumor marker as well as for the marker of inflammation were significantly higher in T4 patients than in the subjects in the T1 + 2 and T3 subgroups as found via the Kruskal–Wallis test. In addition, the differences between CRP levels in T4 (*p* = 0.020), T3 (*p* < 0.0001) and T1 + 2 (*p* < 0.0001) patients were statistically elevated when compared to the control group. There was no significant association between serum CXCR1 levels and tumor size (Table 3). Moreover, we established that serum CXCL1 levels were lower in patients with lymph nodes and distant metastases in comparison to patients without nodal involvement and the presence of distant metastases. Moreover, there was a significant difference between the N0 and N1 subgroups for CXCL1 (*p* = 0.032) levels, which was similar for the CRP (*p* < 0.001) and CEA (*p* = 0.002) concentrations (Table 3). Serum levels of the classical tumor marker were significantly elevated in patients with distant metastases when compared to M0 subjects (*p* = 0.001). However, statistically significant differences in the Dwass–Steele–Critchlow–Fligner test were found between CRP (*p* < 0.001) and CEA (*p* = 0.003) levels in M0 patients and healthy subjects (Table 3). Serum levels of CXCL1 and CXCR1, as well as CEA and CRP concentrations, were elevated in intermediate and high grades (G2 and G3) of CRC in comparison to the low grade (G1) of this tumor; however, the statistical differences were found only for CEA (*p* = 0.005) and CRP (*p* < 0.001) levels via the Kruskal–Wallis test (Table 4).

**Figure 2:**
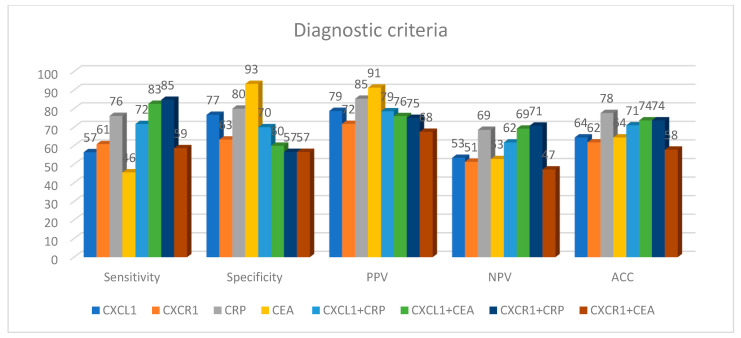
The percentage of elevated concentrations for CXCL1, CXCR1, classic tumor marker (CEA) and C-reactive protein (CRP) in CRC patients. ACC, accuracy; CEA, carcinoembryonic antigen; CRP, C-reactive protein; CXCL1, C-X-C motif ligand 1; CXCR1, C-X-C motif chemokine receptor 1; NPV, negative predictive value; PPV, positive predictive value.

**Figure 3:**
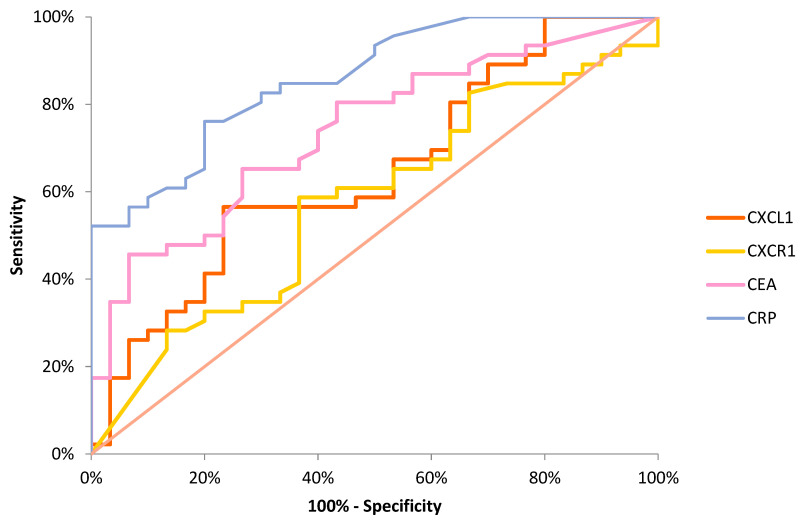
Areas under ROC curves for the measurement of concentrations of analyzed proteins in CRC patients. CEA, carcinoembryonic antigen; CRP, C-reactive protein; CXCL1, C-X-C motif ligand 1; CXCR1, C-X-C motif chemokine receptor 1.

The percentage of elevated concentrations (diagnostic sensitivity) of CXCL1 (57%) and CXCR1 (61%) was higher than that of the classical tumor marker CEA (46%), but lower than that of CRP (76%), whereas it increased in the combined analysis of CXCL1 and CXCR1 with CEA or CRP (Figure 2). The highest value of diagnostic sensitivity was estimated for the combined assessment of CXCR1 with CRP (85%). The diagnostic specificity for CXCL1 (77%) and CXCR1 (63%) was lower in comparison to CRP (80%) and CEA (93%) levels, but was similar to the predictive value for positive (PPV) results. The predictive value for negative (NPV) results for CXCL1 (53%) and CXCR1 (51%) was similar to that of CEA (53%) and lower than that of CRP (69%). The diagnostic accuracy of CXCL1 and CXCR1 was also similar to that of CEA (64%) and lower than that of CRP (78%). The AUC for CXCL1 (0.6449, *p* = 0.0261) was lower than both the AUC for CEA (0.7388, *p* < 0.001) and the AUC for CRP (0.8591, *p* < 0.001). However, the combined assessment of CXCL1 with the classical tumor marker increased the AUC to 0.7674 in the diagnosis of CRC (Figure 3). Furthermore, the AUCs for CXCL1 (0.6173) and CXCR1 (0.6077) were higher than the AUCs for CRP (0.5490) and CEA (0.5769) when differentiating between patients with nodal involvement and patients without nodal involvement. In addition, the AUC for CXCL1 (0.5909) and CXCR1 (0.6364) was lower in comparison to that of CEA (0.6818, *p* = 0.0105) and CRP (0.7557, *p* = 0.0001) in the differentiation between patients with distant metastases and those without distant metastases.

The association between the various risk factors (serum levels of the tested proteins) and the prediction of CRC was first examined through a univariate analysis in order to identify risk factors that qualify for the multivariate model. The results were presented as *p*-values and odd ratios (ORs). We revealed that only the serum levels of CEA (*p* = 0.022, OR = 1.545) and CRP (*p* = 0.003, OR = 1.709) were associated with a significantly increased prediction of CRC. Variables that were found to be statistically significant in the univariate logistic regression analysis were entered into the multivariate model, and then significant variables were removed from the model in a stepwise manner. We established that serum CEA (*p* = 0.027, OR = 1.431) and CRP (*p* = 0.005, OR = 1.661) were significant predictors of CRC risk.

## 4. Discussion

Colorectal cancer (CRC) is the third most commonly diagnosed cancer and the third leading cause of cancer-related death worldwide [39]. It has been proven that chronic inflammation is a prognostic factor for CRC [40]. Moreover, there is growing evidence that an inflammatory tumor microenvironment may promote the development and progression of cancer, including its growth and metastasis [41]. Some clinical investigations have suggested that chemokines and their specific receptors play an important role in tumor development, including CRC. Chemokine (C-X-C motif) ligand 1 (CXCL1) is regulated by multiple signal pathways and the tumor microenvironment, and is associated with tumor growth, cellular transformation, and invasive potential [31,42,43]. This chemokine stimulates chemotaxis and may facilitate the formation of metastases in target organs, particularly in the lungs [44,45,46,47,48].

Increasing evidence has demonstrated that CXCL1 plays a crucial role in the pathogenesis of several malignancies, including CRC. It has been established that CRC stem cells are able to secrete CXCL1 to attract neutrophils, which may promote the tumorigenesis of CRC cells via interleukin-1β. Furthermore, compared with other immune cells, neutrophils are very important for tumor initiation and support early tumorigenesis by inducing angiogenesis via metalloproteinase-9 [49,50]. Thus, finding novel biomarkers useful in the diagnosis and prognosis of CRC is of utmost importance.

There are several lines of evidence that prove the significance of selected chemokines and their receptors in tumor pathogenesis, including CRC. However, most of the research conducted to date has been performed on CRC tissue using time-consuming immunohistochemical methods [5,7,22,45,46]. As far as we know, there have been no studies assessing serum levels of CXCL1 and CXCR1 in CRC patients in relation to the clinicopathological features of the tumor or evaluating the prognostic and diagnostic utility of CXCL1 and CXCR1 in comparison to the well-established classical tumor biomarker (CEA) and the indicator of the inflammatory process (CRP). Furthermore, the present study is a continuation of our most recent investigations aimed at establishing new biochemical markers for CRC [26,27] and other studies exploring the diagnostic significance of various chemokines and their receptors in other malignancies of the digestive tract [35,36,37,38].

Our present study revealed that CXCL1 concentrations were significantly higher in CRC patients when compared to healthy controls, and were similar to those of the classical tumor biomarker CEA and the marker of inflammation CRP. These findings are consistent with the data published by other authors who demonstrated that the level of serum CXCL1 in CRC patients is higher than in healthy donors [44]. Investigations performed on the sera of CRC patients using the ELISA method and confirmed by the immunohistochemical method also demonstrated that CXCL1 expression is significantly higher in CRC tissues than in normal tissues [33]. Based on our results and the findings of other authors, we concluded that CRC cells are able to synthesize this chemokine. Other studies have also revealed that 79% of CRC cells show positive immunoreactivity for CXCL1 [31]. Furthermore, Li et al. evaluated the constitutive expression of CXCL1 in human colon carcinoma cells with different metastatic potentials and revealed that non- and low metastatic cells express lower levels of CXCL1 when compared to highly metastatic CRC [50]. The authors concluded that the constitutive CXCL1 expression is correlated with a more invasive phenotype [50,51].

We found that the concentrations of CXCL1 and CEA were higher in patients with adenocarcinoma than in those with mucinous adenocarcinoma, and these differences were significant only when compared to the control group. In addition, the serum levels of CXCL1 and CXCR1 as well as the CEA and CRP concentrations were higher in high and intermediate grades of CRC in comparison to low-grade CRC. A study by Oladipo et al. also revealed no significant correlation between CXCL1 and CXCR1 expression and tumor stage or grade [52].

In the present paper, the concentrations of CXCL1 were significantly correlated with CRP levels, which was similarly true for the concentrations of CEA. We previously established significant correlations between the presence of distant metastases, CEA and CRP levels, and serum concentrations of another CXC chemokine—CXCL8 [28]. However, the Spearman rank correlation test did not show any correlations between serum CXCR2 concentrations and the clinicopathological features of CRC [29].

The present paper shows that serum CEA and CRP levels are significant predictors of CRC risk. Similar findings were reported in our previous study in which we demonstrated that serum CXCL8, CRP and CEA, but not CXCR2, were the only significant predictors of CRC risk [28,29].

The present study is the first evaluation of the diagnostic usefulness of CXCL1 and CXCR1 in relation to the well-known CRC biomarker CEA. The diagnostic sensitivity of CXCL1 and CXCR1 was higher than that of the classical tumor marker and increased in the combined analysis with CEA. The highest value of diagnostic sensitivity was estimated for the combined assessment of CXCL1 with CEA and CXCR1 with CRP. Moreover, in the present paper, the diagnostic specificity and PPV results for CXCL1 levels were lower in comparison to CRP and CEA levels, while NPV results for CXCL1 were the same as for CEA and lower than for CRP, and similar results were found for diagnostic accuracy. The AUC for CXCL1 was marginally lower than the AUC for CEA in the diagnosis of CRC. Based on the analysis of the percentage of elevated concentrations, we indicated that the combined analysis of CXCL1 and CXCR1 with the classical tumor marker might be more useful in the diagnosis of CRC in comparison to the measurement of a single biomarker. In our previous publications regarding the significance of a specific receptor for CXCL1, CXCR2, as well as its other ligand, CXCL8, in CRC, we revealed that diagnostic sensitivity, NPV and accuracy were higher for serum CXCL8 when compared to CEA. Moreover, the AUC for CXCL-8 was higher than the AUC for CEA in CRC patients. In addition, the diagnostic sensitivity of CXCR2 was higher compared to that of CEA and increased in the combined analysis of CXCR2 with CEA [28,29]. A similar relationship between the receptor of the chemokine and CEA was found in the present study. Furthermore, we can conclude that serum CXCL8 might be a better biochemical marker candidate in the diagnosis of CRC than the classical tumor marker used in routine clinical practice. However, a limitation of our study was the small number of cancer patients. The study and control groups will be expanded in our future studies. The second limitation concerns the nature of chemokines that play a role in physiological as well as pathological conditions, including CRC. In addition, the majority of cases in the study group involved patients in stages T1, T2 and T3 as well as M0, without the presence of distant metastases.

## 5. Conclusions

There is a lack of research on novel biomarkers of CRC since the early diagnosis of this malignancy through the assessment of serum biochemical markers remains a great challenge. To our knowledge, this is the first study evaluating the diagnostic utility of serum CXCL1 and CXCR1 in CRC patients in relation to the well-established tumor biomarker (CEA) and the marker of inflammation (CRP). According to the presented findings, we conclude that serum CXCL1 and CXCR1 might be useful in the diagnosis of CRC, particularly in the combined analysis with CEA. In addition, among all proteins tested, serum CXCL1 seems to be the best indicator in the differentiation between CRC patients with nodal involvement and patients without the presence of lymph node metastasis. Although a number of investigations in animal models have demonstrated that the CXCL1/CXCR2 and CXCL8/CXCR1 axes promote tumor growth and angiogenesis of several cancer types, including CRC, CXCL1′s significance in human CRC needs to be clarified before this chemokine may be used as a biomarker for this malignancy. However, the results of this work represent an important step toward increasing CRC detection. This is extremely important in the era of rapid socio-economic development, as the number of CRC cases increases with the level of development in the country (due to factors such as high consumption of red meat and obesity). Actions aimed at detecting new parameters are extremely important, as they will indicate the initiation of neoplastic changes and their progression or recurrence in a quick and minimally invasive way.

## Figures and Tables

**Table 1:** Characteristics of patients with colorectal cancer (CRC).

Tested Variable		Number of Patients
Group	Colorectal cancer (CRC) patients	46
Gender	Male	30
	Female	16
TNM stage	I	14
	II	12
	III + IV	20
Depth of tumor invasion (T factor)	T1 + 2	19
	T3	23
	T4	4
Nodal involvement (N factor)	Node-negative	26
	Node-positive	20
Distant metastases (M factor)	M0	44
	M1	2
Histological subtypes of tumor	Adenocarcinomas	36
	Mucinous adenocarcinomas	10

CRC, colorectal cancer; G, tumor grade; M, distant metastases; N, nodal involvement; T, depth of tumor invasion; TNM, tumor–node–metastasis.

**Table 2:** Serum concentrations of CXCL1, CXCR1, CEA and CRP in relation to tumor stage in CRC patients.

		CXCL1 (pg/mL)			CXCR1 (ng/mL)			CEA (ng/mL)			CRP (mg/L)		
TNM		Min	Me	Max	Min	Me	Max	Min	Me	Max	Min	Me	Max
I		58.48	125.51	514.64	0.00	0.05	0.66	0.5	2.01	31.13	1.3	3.8	77.2
II		71.88	156.66	264.83	0.00	0.03	0.29	1.13	1.83	12.17	1.2	12.15	65.2
III + IV		61.72	91.96	390.99	0.00	0.02	0.73	0.5	2.94	1176.5	1.0	4.8	83.6
Control Group (CG)		45.31	97.45	442.18	0.00	0.04	0.18	0.5	1.05	7.82	0.2	1.2	5.8
*p* *		0.068			0.392			0.004			<0.0001		
*p* **	I vs. II	0.984			0.949			0.99			0.67		
	I vs. III + IV	0.835			0.612			0.827			1.00		
	I vs. CG	0.191			1.000			0.259			0.001		
	II vs. III + IV	0.474			0.834			0.931			0.742		
	II vs. CG	0.068			0.863			0.032			<0.0001		
	III + IV vs. CG	0.914			0.400			0.009			0.001		

* Statistically significant when *p* < 0.05 in Kruskal–Wallis test; ** statistically significant when *p* < 0.05 in Dwass–Steele–Critchlow–Fligner test. CEA, carcinoembryonic antigen; CRP, C-reactive protein; CRC, colorectal cancer; CXCL, C-X-C motif ligand 1; CXCR1, C-X-C motif chemokine receptor 1; M, distant metastases; Max, maximum; Me, median; Min, minimum; N, nodal involvement; T, depth of tumor invasion; TNM, tumor–node–metastasis.

**Table 3:** Serum concentrations of CXCL1, CXCR1, CEA, and CRP in relation to clinicopathological characteristics of CRC.

		CXCL1 (pg/mL)			CXCR1 (ng/mL)			CEA (ng/mL)			CRP (mg/L)		
		Min	Me	Max	Min	Me	Max	Min	Me	Max	Min	Me	Max
T3		61.72	130.38	298.97	0.00	0.03	0.73	0.50	1.99	1176.50	1.00	6.40	83.60
T4		62.14	105.93	165.34	0.00	0.03	0.07	1.14	2.86	5.57	2.50	20.60	33.30
T1 + 2		58.48	124.70	514.64	0.00	0.03	0.66	0.50	1.87	31.13	1.30	3.70	77.20
Control Group (CG)		45.31	97.45	442.18	0.00	0.04	0.18	0.50	1.05	7.82	0.20	1.20	5.80
*p* *		0.163			0.720			0.005			<0.0001		
*p* **	3 vs. 4	0.928			0.997			1.000			0.694		
	3 vs. 1 + 2	0.976			1.000			0.904			0.986		
	3 vs. CG	0.321			0.730			0.007			<0.0001		
	4 vs. 1 + 2	0.885			0.988			0.970			0.692		
	4 vs. CG	0.982			0.853			0.174			0.020		
	1 + 2 vs. CG	0.184			0.916			0.093			<0.0001		
Node-negative		58.48	130.07	514.64	0.00	0.04	0.66	0.50	1.86	31.13	1.20	6.90	77.20
Node-positive		61.72	91.96	390.99	0.00	0.02	0.73	0.50	2.94	1176.50	1.00	4.80	83.60
CG		45.31	97.45	442.18	0.00	0.04	0.18	0.50	1.05	7.82	0.20	1.20	5.80
*p* *		0.032			0.261			0.002			<0.0001		
*p* **	Node-negative vs. Node-positive	0.367			0.424			0.649			0.839		
	Node-negative vs. CG	0.017			0.908			0.014			<0.0001		
	Node-positive vs. CG	0.790			0.262			0.005			<0.0001		
M0		58.48	125.51	514.64	0.00	0.03	0.73	0.50	1.90	1176.50	1.00	6.85	83.60
M1		85.53	106.13	126.73	0.03	0.05	0.07	3.66	3.77	3.87	2.00	2.25	2.50
Control Group (CG)		45.31	97.45	442.18	0.00	0.04	0.18	0.50	1.05	7.82	0.20	1.20	5.80
*p* *		0.096			0.466			0.001			<0.0001		
*p* **	0 vs. 1	0.903			0.792			0.664			0.446		
	0 vs. CG	0.079			0.483			0.003			<0.0001		
	1 vs. CG	0.948			1.00			0.073			0.403		

* Statistically significant when *p* < 0.05 in Kruskal–Wallis test; ** statistically significant when *p* < 0.05 in Dwass–Steele–Critchlow–Fligner test. CEA, carcinoembryonic antigen; CG, control group; CRC, colorectal cancer; CRP, C-reactive protein; CXCL1, C-X-C motif ligand 1; CXCR1, C-X-C motif chemokine receptor 1; M, distant metastases; Max, maximum; Me, median; Min, minimum; N, nodal involvement; T, depth of tumor invasion; TNM, tumor–node–metastasis.

**Table 4:** Serum concentrations of CXCL1, CXCR1, CEA, and CRP in relation to tumor grade and histological subtype of CRC.

		CXCL1 (pg/mL)			CXCR1 (ng/mL)			CEA (ng/mL)			CRP (mg/L)		
		Min	Me	Max	Min	Me	Max	Min	Me	Max	Min	Me	Max
Control group (CG)		45.31	97.45	442.18	0.00	0.04	0.18	0.50	1.05	7.82	0.20	1.20	5.80
G1		65.97	71.08	119.23	0.00	0.05	0.07	1.23	1.31	7.96	1.10	1.80	22.60
G2		58.48	129.77	514.64	0.00	0.03	0.73	0.50	1.99	1176.50	1.00	6.40	83.60
G3		94.82	126.73	162.27	0.07	0.09	0.09	1.11	3.87	4.69	2.50	16.10	25.80
*p* *		0.063			0.352			0.005			<0.0001		
*p* **	CG vs. G1	0.982			0.982			0.511			0.572		
	CG vs. G2	0.095			0.625			0.004			<0.0001		
	CG vs. G3	0.673			0.816			0.235			0.069		
	G1 vs. G2	0.332			0.989			0.996			0.803		
	G1 vs. G3	0.421			0.421			0.996			0.695		
	G2 vs. G3	0.999			0.423			0.996			0.950		
Control group (CG)		45.31	97.45	442.18	0.00	0.04	0.18	0.50	1.05	7.82	0.20	1.20	5.80
Adenocarcinomas (A)		58.48	126.53	514.64	0.00	0.03	0.73	0.50	2.07	72.26	1.00	4.40	83.60
Mucinous adenocarcinomas (MA)		61.72	95.99	211.48	0.00	0.01	0.17	1.02	1.87	1176.50	1.00	7.60	65.20
*p* *		0.045			0.329			0.002			<0.0001		
*p* **	CG vs. A	0.040			0.730			0.004			<0.0001		
	CG vs. MA	0.937			0.328			0.025			0.001		
	A vs. MA	0.407			0.556			0.994			0.921		

* Statistically significant when *p* < 0.05 in Kruskal–Wallis test; ** statistically significant when *p* < 0.05 in Dwass–Steele–Critchlow–Fligner test. CEA, carcinoembryonic antigen; CRC, colorectal cancer; CRP, C-reactive protein; CXCL1, C-X-C motif ligand 1; CXCR1, C-X-C motif chemokine receptor 1; G, tumor grade; Max, maximum; Me, median; Min, minimum. The Spearman rank test was used to perform correlation analyses. CXCL1 concentrations were significantly correlated with CRP (*p* = 0.001) and CXCR1 (*p* = 0.040), while CEA levels were significantly associated with CRP levels (*p* = 0.009) (Table 5).

**Table 5:** Correlations between clinicopathological characteristics of CRC and serum concentrations of tested proteins.

		T	N	TNM	CXCL1	CXCR1	CEA	CRP
T	r	1.00	0.32	0.60	−0.10	−0.01	0.10	0.16
	*p*		0.029 ^a^	<0.0001 ^a^	0.521	0.971	0.515	0.297
N	r	0.32	1.00	0.87	−0.13	−0.11	0.09	−0.06
	*p*	0.029 ^a^		<0.0001 ^a^	0.407	0.452	0.538	0.686
TNM	r	0.60	0.87	1.00	−0.16	−0.17	0.15	−0.04
	*p*	<0.0001 ^a^	<0.0001 ^a^		0.293	0.255	0.317	0.786
CXCL1	r	−0.10	−0.13	−0.16	1.00	0.24	0.13	0.36
	*p*	0.521	0.407	0.29		0.040 ^a^	0.268	0.001 ^a^
CXCR1	r	−0.01	−0.11	−0.17	0.24	1.00	0.08	−0.01
	*p*	0.971	0.452	0.25	0.040 ^a^		0.481	0.924
CEA	r	0.10	0.09	0.15	0.13	0.08	1.00	0.30
	*p*	0.515	0.538	0.32	0.268	0.481		0.009 ^a^
CRP	r	0.16	−0.06	−0.04	0.36	−0.01	0.30	1.00
	*p*	0.297	0.686	0.79	0.001 ^a^	0.924	0.009 ^a^	

^a^ Statistically significant when *p* < 0.05; CEA, carcinoembryonic antigen; CRP, C-reactive protein; CXCL1, C-X-C motif ligand 1; CXCR1, C-X-C motif chemokine receptor 1; M, distant metastases; N, nodal involvement; T, depth of tumor invasion; TNM, tumor–node–metastasis.

## Data Availability

The data that support the findings will be available on request under the corresponding author’s e-mail.

## Supplementary Materials

The following supporting information can be downloaded at: https://www.mdpi.com/article/10.3390/biomedicines11071933/s1.
